# Case of Anasarca

**Published:** 1840

**Authors:** 


### CASE OF ANASARCA.

Dr. Millikin of Courtland, Alabama, has transmitted to us the notes of a case of anasarca, apparently of malarious origin, and very inveterate in its character. We do not think it entitled to publication in detail, but derive from it two clinical facts, which, although not new, are worthy of being noticed. *First.* The patient was *twice* subjected to a salivation, but without any other effect, than that of prostrating her strength. *Second.* She was cured by the persevering use, three times aday, of the following, *non*-mercurial, *recipe*.

℞ aloet. socot. pulv. gr. j, pulv. jalap. gr. ij. scil. maritim. gr. j. Mix and make one pill.

During the use of this simple compound the patient drank freely of a cold infusion of slippery elm bark, and a decoction of elder blossoms alternately. At the close, the debility was successfully met with vegetable bitters and mineral acids. D.

